# New ways of patient participation in clinical and translational cancer research

**DOI:** 10.1007/s10585-026-10450-w

**Published:** 2026-11-12

**Authors:** Wiebke Kragl, Jonathan P. Sleeman, Jörg Haier

**Affiliations:** 1 OnkoBeirat Comprehensive Cancer Center M-V, Rostock, Germany; 2 Comprehensive Cancer Center M-V, University Medicine Rostock, Schillingallee 68, 18057 Rostock, Germany; 3 Medical Faculty Mannheim, University of Heidelberg, Heidelberg, Germany

**Keywords:** Patient participation, Cancer research, Acceptance, Patient representatives, Decision making

Clinical and translational cancer research is rarely possible without patient participation. This participation can take many forms. For example, patients and as-yet unaffected individuals contribute biological samples for research. The use of data drawn from patients or population groups relevant to cancer enables epidemiological statistical analysis. Patients also enroll as participants in clinical investigator initiated or industry-driven trials. There are also a variety of other ways in which patients engage, be that through providing other forms of data, information or participation. Thus, numerous interfacing relationships exist between researchers, scientists, patients and affected population. This is particularly true in the field of metastasis research.

Despite the importance of patient participation in cancer and metastasis research, one particular dimension of patient participation that has been recognized in recent years as being critically important remains insufficiently integrated in research projects, namely the active participation of patients or other non-medical stakeholders in the definition of research questions, intended outcomes or endpoints in clinical and translational projects. This means that the most affected stakeholder group continues to have only limited possibilities for participating in quality control, and in particular is frequently unable to integrate patient-related content into the structure and processes of research projects, or to help steer the design of the project or its overall direction. Accordingly, many stakeholders have recently acknowledged the importance of shifting the role of patients in cancer research away from being regarded as merely research subjects to becoming active contributors or even co-workers in research projects.

**Fig. 1:**
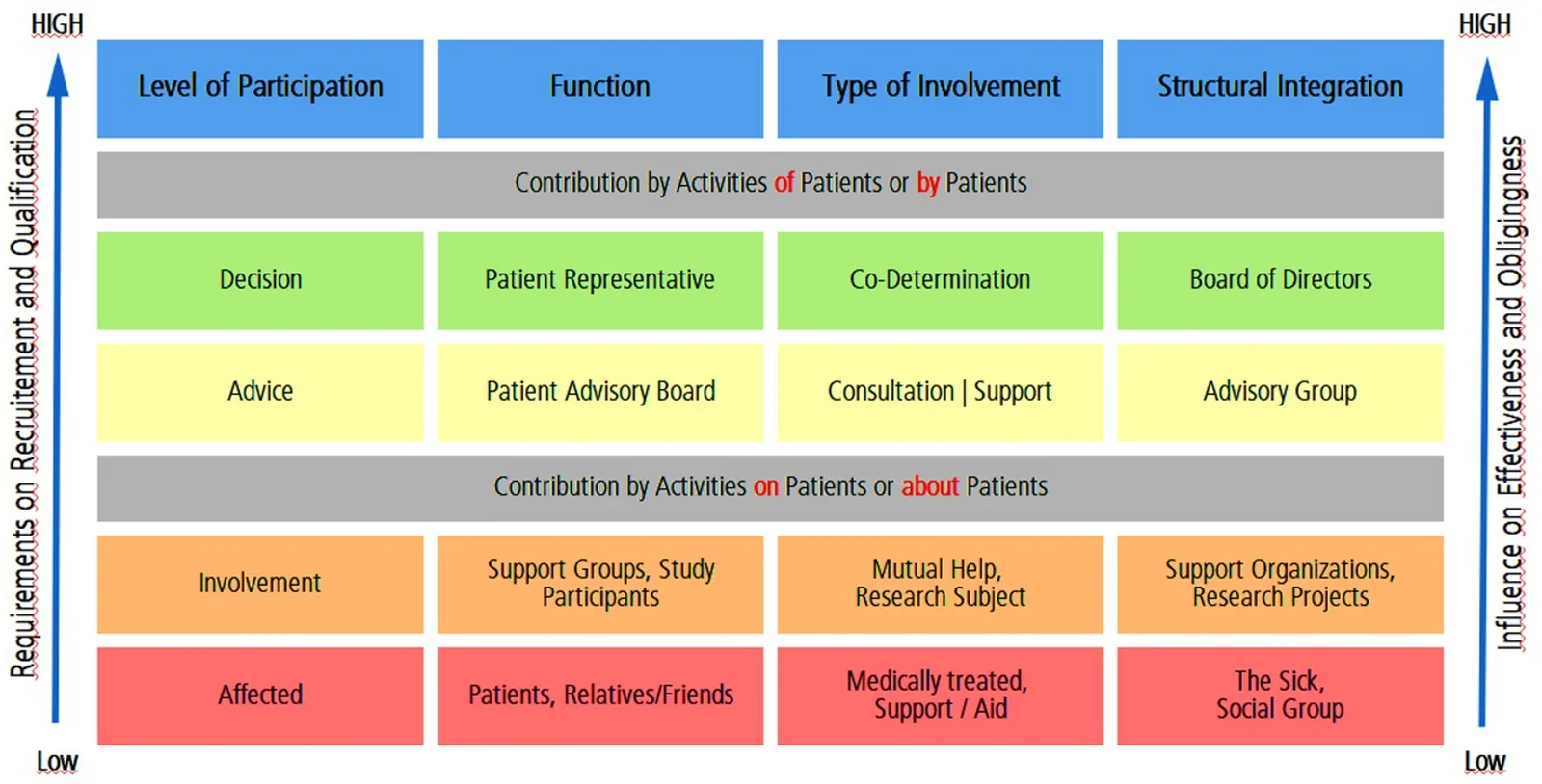
Different roles of patients in clinical and translational cancer research and levels of implementation

Figure 1 illustrates the most important levels of engagement for patients who are interested in taking a more active role in contributing to the medical and research system. Starting at the bottom of the table with passive participation, increasingly active levels of involvement can be implemented. Some of these are already available to patients and/or their relatives in cancer care institutions, or are under development or in the planning phase. As involvement increases at each level, so do the requirements in terms of the qualifications and personal skill sets of those who are recruited for these roles. On the other hand, each level of participation also increases the potential effectiveness of the Patient Representatives and Patient Experts involved to voice their own perspectives, to defend their contributions and ideally to vote within development of research strategies. However, in the real-world practice of cancer research, these contributions are frequently only regarded as a theoretical proposal of participation, such as the mere provision of Letters of Intent for funding applications. Acceptance of higher levels of patient participation in clinical and translational research by medical professionals and scientists is often limited or treated as an external request, resulting in limited active and comprehensive inclusion.

While the recruitment of patients to participate in trials is standard practice in many institutions, the full empowerment of patient experts requires targeted activities and frequent consultation with decision-makers in clinical and translational science. Effective patient participation is usually not integrated into decision-making processes, or into the legal and financial framework of cancer care providers. For example, voluntary patient representatives should be integrated into decisional structures, such as in the Board of Directors of cancer centres and/or accompanying advisory boards. This may encompass different age groups and cancer entities, as well as perspectives from involved relatives of cancer patients. Their integration provides serious challenges to both the voluntary patients occupying these new roles, and to the medical/scientific professionals who have to integrate them into their established structures and pathways. While the former have to learn their new role, often with the initial challenge to identify and define it, the latter have to change their attitudes and to make an effort to integrate these new partners into their daily work. Therefore, a co-creation and sustainable joint implementation plan of a patient participation initiative should be combined with mission/vision statements and a multi-year action plan designed by both research professionals and patient experts/representative.

In the near future, medical laypersons will become part of research projects not only as subjects of research, but also as participants in the research process itself. There is broad consensus^1^. that the integration of patient experts into the definition and execution of research projects will benefit everyone concerned. Research projects are expected to become more relevant for patients and their own decision making. The dedicated involvement of patients’ perspectives into scientific objectives will enable a better prioritization of the practical relevance and usability of research goals and results. However, there is only limited experience and very little established practice regarding how patients’ perspectives can be integrated into the conceptualization, execution and discussion of research projects and results without interfering with the freedom of research. In particular, there is still an ongoing debate as to whether the patients (and their relatives) should be kept in a lay-person status rather than an expert status. Frequently, discussions about patient involvement in research related decision making, such as in steering bords, argue that patients do not have all required expertise for clinical and translational research. However, the same is true for the professional participants: Researchers provide scientific expertise, clinicians have medical expertise and patients integrate another form of expertise: lived experience that is usually missing in researchers and clinicians. These differences in fields of expertise may result in difficulties for the patient experts to sufficiently express their arguments. A potential urge to conform, for example with the perspective of the clinicians, must be counteracted, and this can be achieved through thorough training and by ultimately encouraging patients to lead to self-management of the participation initiative.

A number of questions and challenges must be addressed during the implementation of this integration:

- Which role can medical laypersons fulfil in a research project? For example, patients may act as subproject managers in research projects, and even serve as Patient Representatives, acting as indispensable partners within the steering committee of larger projects.
- What qualifications can or should be required of patients who contribute to cancer research in this novel role? No standards or mandatory requirements currently exist, and the role needs to be further defined and established. Currently, this is in the sole discretion of the professionals staffing the project. However, dedicated educational programs should be developed with continuous improvement following initial successful pilot initiatives^2^. The development and implementation of these concepts also needs needs the obligatory consideration of their perspectives. The exchange of these curricula, educational materials and experiences should be initiated by the broad cancer patient community and their supporters, such as via the SPARC initiative for personalized cancer medicine^1^.
- How should the resources that patients invest into a research project be compensated? This requires balancing adequate reimbursement against the risk of biasing incentives, while also accounting for special circumstances, such as individual disabilities.
- How can we ensure a sufficient consideration if the patient’s perspective conflicts with that of the professionals in the room?

Finally, similar to the integration of patient participation into clinical processes and structures, it is to be expected that the involvement of patients in steering committees or project management teams will be challenging for all affected stakeholders. Patients are new to the structures, processes and social dynamics of research projects and have to find their place within these teams, while professional researchers may be uncertain how to engage with this new type of team member, and how to respond to their ideas and proposals. Resistance to establishing new operating procedures may leave research objectives or the interpretation of results controversial, and rules for interaction will likely need to be standardized.

Patient participation in cancer and metastasis research holds significant potential. However, a steep learning curve lies ahead, and the processes and structures needed to effectively integrate patients and their perspectives into cancer research are at an early stage of development. Preparing patient volunteers to participate effectively in cancer research will be a key requirement in the near future.

## Data Availability

No datasets were generated or analysed during the current study.

## Footnotes

1 European Joint Action Program:SPARC — Support ofPersonalized Medicine Approaches in Cancer; https://sparc-project.eu/index.html#about.

2 Patient Expert Academy for Tumor Diseases (PEAK), NationalCenter for Tumor Diseases (NCT),Germany; https://nct.dkfz.de/en/patient-involvement/patient-expert-academy.html.

